# Impact of Nb_2_O_5_ Coating Produced by Using the Reactive Sputtering Technique on Bacterial Biofilm Formation

**DOI:** 10.1021/acsomega.5c10066

**Published:** 2026-01-20

**Authors:** Alessandro Márcio Hakme da Silva, Alessandra Baptista, Valeska Bezerra Santana Albuquerque, Josué de Moraes, Carlos Alberto Fortulan, Mariana Amorim Fraga, Rogério Valentim Gelamo, Ricardo Scarparo Navarro, Jéferson Aparecido Moreto

**Affiliations:** † Scientific and Technological Institute, Bioengineering Graduate Program, 149945 Brazil University, São Paulo, SP 08230-030, Brazil; ‡ Research Center for Neglected Diseases, Centro de Pós Graduação e Pesquisa, Guarulhos, 92928 Guarulhos University, Guarulhos, SP 07023-070, Brazil; § Mechanical Engineering Department, São Carlos School of Engineering, University of São Paulo (USP), São Carlos, SP 13566-590, Brazil; ∥ 42524 Mackenzie Presbyterian University, São Paulo, SP 01302-907, Brazil; ⊥ Institute of Technological and Exact Sciences, 74348 Federal University of Triângulo Mineiro (UFTM), Uberaba, MG 38025-180, Brazil; # Materials Engineering Department, São Carlos School of Engineering, University of São Paulo (USP), São Carlos, SP 13566-590, Brazil

## Abstract

The reactive sputtering technique has been employed to deposit niobium pentoxide (Nb_2_O_5_) thin films onto the surfaces of the Ti-6Al-4 V alloy, which is widely used in trauma care and tissue repair. This approach has shown significant potential in enhancing the alloy’s resistance to uniform and localized corrosion, as well as improving its wear and fatigue performance. In this study, Nb_2_O_5_ thin films were deposited on Ti-6Al-4 V surfaces using reactive DC sputtering, and their biofilm-modulating effects were evaluated in the presence of artificial saliva (AS) and two clinically relevant bacteria strains—*Staphylococcus aureus* ATCC 25923 (Gram-positive) and *Escherichia coli* ATCC 25922 (Gram-negative). The extent of biofilm coverage, expressed as a percentage, was quantitatively assessed using scanning electron microscopy (SEM) coupled with energy-dispersive spectroscopy (EDS). This combined analytical approach allowed for detailed morphological examination of the biofilm’s distribution. Results demonstrated that the uncoated Ti-6Al-4 V surfaces exhibited 99.83% organic retention after saliva exposure and up to 74.94% biofilm coverage with *E. coli*, while Ti-6Al-4 V/Nb_2_O_5_ specimens showed lower retention under the same conditions (85.11 and 51.10%, respectively). Notably, *S. aureus* adhesion was markedly reduced on the coated samples (67.42%) when compared to that on the AS sample (40.68%), suggesting species-specific modulation of bacterial colonization. These findings indicate that Nb_2_O_5_ coatings can alter the surface wettability and biofilm architecture, reducing nonspecific organic adsorption and selectively influencing bacterial adhesion. This study underscored the potential of Nb_2_O_5_ coatings for the development of multifunctional biomedical surfaces exhibiting both antimicrobial and biointeractive properties.

## Graphical abstract

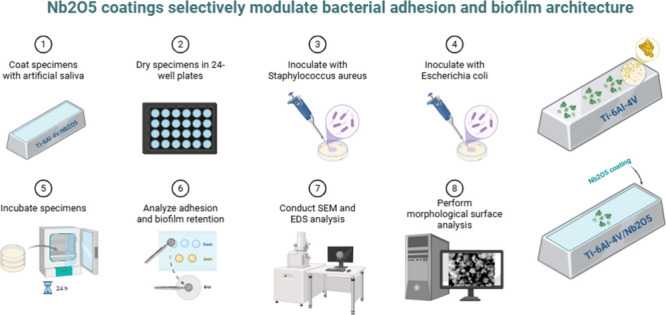

## Introduction

Ti-6Al-4 V alloy is widely used across various industrial sectors owing to its exceptional strength-to-weight ratio, corrosion resistance to uniform and localized processes, and biocompatibility.^1^ In biomedical applications, it is a key material for orthopedic implants (e.g., hip and knee prostheses, bone plates), dental implants, and other medical devices that require durability and physiological compatibility.^2,3^

Nb_2_O_5_ coating has garnered considerable attention in biomaterial research owing to its remarkable thermal and chemical stability, exceptional resistance to wear and corrosion, bioactivity, and outstanding biocompatibility.^4^ Studies have consistently highlighted the beneficial role of Nb_2_O_5_ in biomedical surface coatings, particularly in improving antibacterial performance and surface biofunctionality.^5,6^ Recent research has shown that functionalized coatings on Ti-6Al-4 V can significantly reduce bacterial adhesion, including *Streptococcus mutans* and *Escherichia coli*, which are critical factors in preventing biofilm formation in dental applications.^6^

Recent efforts have concentrated on the development of a nanostructured Nb_2_O_5_ coating using reactive magnetron sputtering. When applied to Ti-6Al-4 V, the Nb_2_O_5_ coating significantly enhances uniform and localized corrosion resistance and mechanical integrity (including both wear and fatigue), as well as biofunctional properties.^7–11^ In addition to mitigating inflammatory responses, Nb_2_O_5_ coating promotes cellular compatibility, positioning them as promising candidates for biomedical applications.^8–10^ The global relevance of this research is further highlighted by Brazil’s dominance in niobium production (90% of reserves, with 80% sourced from the Barreiro mine alone),^12^ which ensures material availability for scalable applications.

Despite these advancements, the species-specific effects of the Nb_2_O_5_ coating on bacterial adhesion, particularly under physiologically relevant conditions, remain poorly understood. This knowledge gap necessitates further investigation to elucidate how different bacterial species interact with Nb_2_O_5_-coated surfaces. This study evaluates Nb_2_O_5_-coated Ti-6Al-4 V surfaces for their ability to resist biofilm formation by *S. aureus* (Gram-positive) and *E. coli* (Gram-negative) in artificial saliva (AS), a medium that mimics the ionic/protein composition of human saliva.^13,14^ AS provides a clinically representative environment due to its lubricating properties and support for microbial adhesion.^15^ *S. aureus* was selected for its role in implant-associated infections,^15^ while *E. coli* serves as a model Gram-negative biofilm former.^16^ By quantifying bacterial retention and organic adsorption, this study aimed to elucidate how Nb_2_O_5_ coatings modulate surface-biofilm interactions,^17^ thereby providing valuable insights for the design of infection-resistant implants.^18,19^ Such an understanding is essential for developing biomaterials that minimize bacterial adhesion and enhance the long-term functionality and safety of biomedical devices in clinical environments. These findings may enable innovative surface engineering strategies^18–20^ that effectively resist biofilm formation and substantially reduce postoperative infection risks.^21^

## Results

### Morphological Surface Analyses by Scanning Electron Microscopy (SEM)

A qualitative SEM assessment was conducted on the surfaces of Ti-6Al-4 V and Ti-6Al-4 V/Nb_2_O_5_ under different conditions, focusing on micromorphological features such as surface irregularities, contrast distribution, homogeneity, and signs of microbial retention. At a magnification of 3000 (5 μm), the uncoated Ti-6Al-4 V (Figure 1A) exhibits a comparatively smooth, continuous contrast, whereas Ti-6Al-4 V/Nb_2_O_5_ (Figure 1B) shows a more heterogeneous grayscale with discernible grooves. After exposure to AS (Figure 1C,D), both substrates display a film-like, low-contrast layer. On Ti-6Al-4 V/Nb_2_O_5_, the coverage appears laterally more uniform, as indicated by the continuity of the grayscale and fewer boundary discontinuities. The interpretation of these findings is restricted to morphology and will be contextualized later with wettability and surface energy measurements.

**Figure 1:**
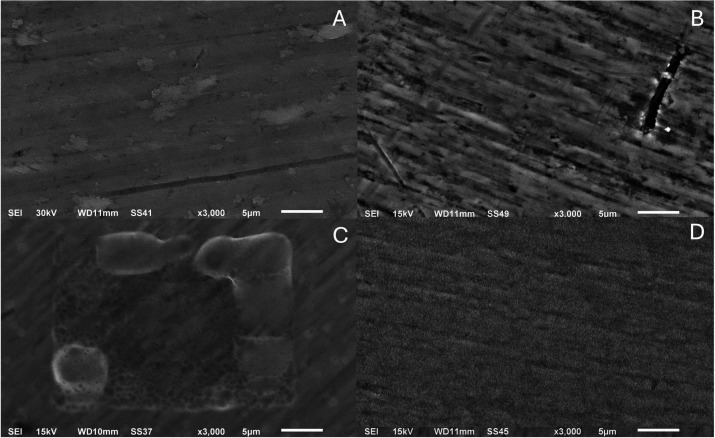
SEM micrographs of the samples: (A) Ti-6Al-4 V, (B) Ti-6Al-4 V/Nb_2_O_5_, (C) Ti-6Al-4 V (AS), and (D) Ti-6Al-4 V/Nb_2_O_5_ (AS) (×3000, 5 μm).

At a magnification of 3000 (5 μm) without saliva preconditioning, Ti-6Al-4 V (Figure 2A) and Ti-6Al-4 V/Nb_2_O_5_ (Figure 2B) exhibit distinct adhesion patterns. Adherent cocci, consistent with *S. aureus*, form localized aggregates, whereas rod-shaped cells consistent with *E. coli* appear more laterally dispersed (Figure 2C,D); arrows/insets mark representative cells, and diffuse low-contrast deposits suggest extracellular material. Within the field of view, the coated alloy showed more continuous lateral coverage. Interpretation is restricted to surface morphology; quantitative surface coverage values are reported in Table 3, and EDS analysis of surface composition.

**Figure 2:**
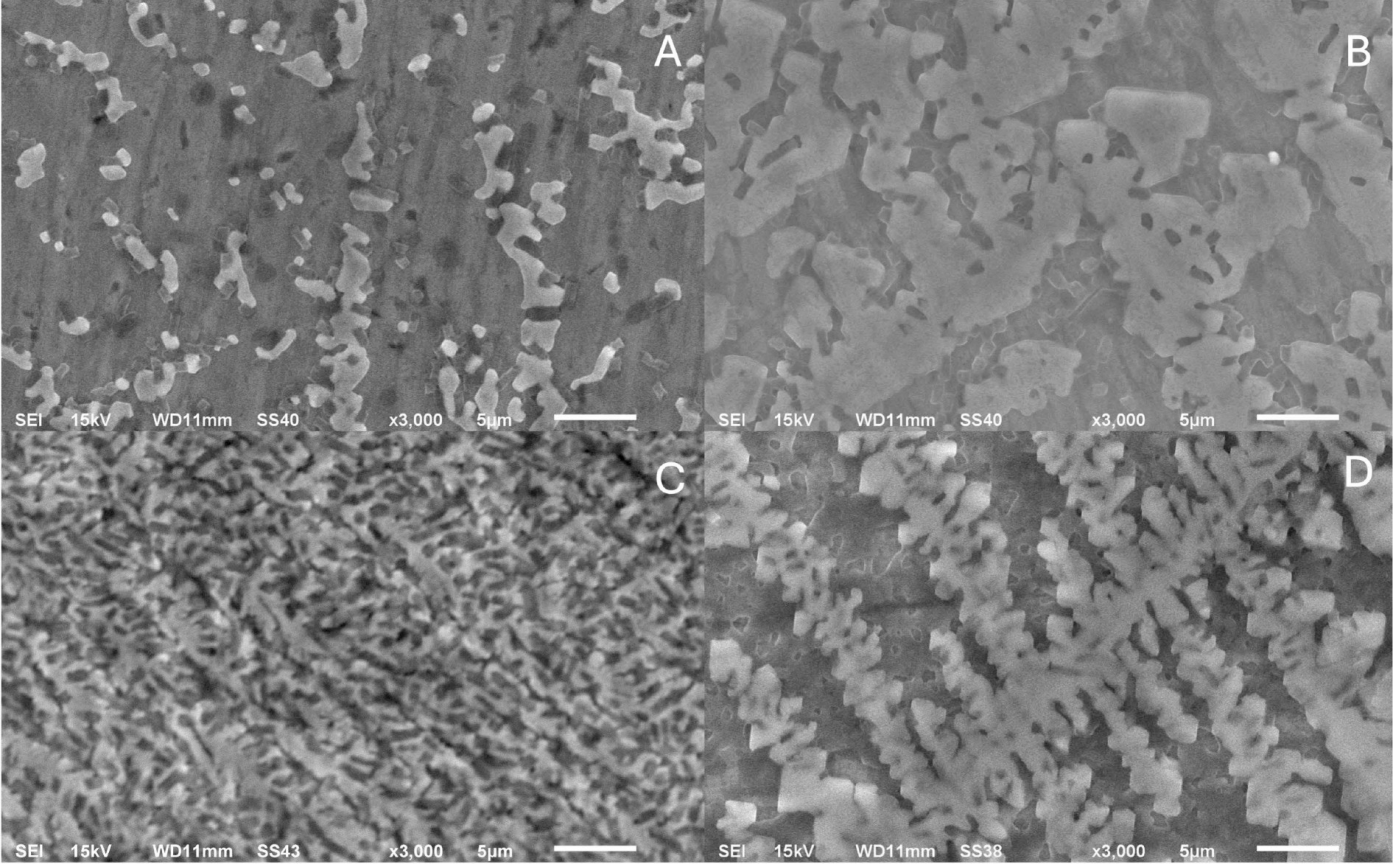
SEM micrographs of the samples: (A) Ti-6Al-4 V (*S. aureus*), (B) Ti-6Al-4 V/Nb_2_O_5_ (*S. aureus*), (C) Ti-6Al-4 V (*E. coli*), and (D) Ti-6Al-4 V/Nb_2_O_5_ (*E. coli*) (×3000, 5 μm).

At a magnification of 3000 (5 μm) in Figure 3, after AS conditioning, both substrates display a continuous, low-contrast background compatible with a pellicle, upon which cocci consistent with *S. aureus* (Figure 3A,B) or rod-shaped cells consistent with *E. coli* (Figure 3C,D) are observed. Relative to the unconditioned state (Figure 2), adherent biomass within the field appears more continuous, with *S. aureus* forming compact, localized clusters and *E. coli* showing thinner, laterally spread deposits. Interpretation is restricted to surface morphology; EDS analysis of surface composition and quantitative coverage metrics is reported in Table 3. These patterns are consistent with the wettability/surface free energy shift measured for Nb_2_O_5_, while AFM indicates that the nanoscale roughness remains unchanged.

**Figure 3:**
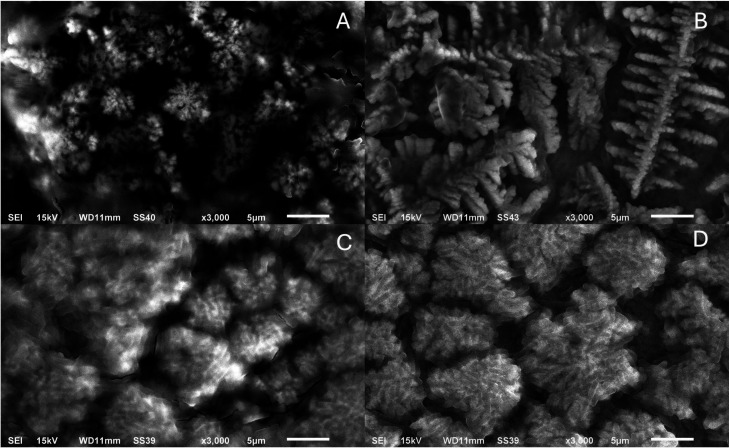
SEM micrographs of the samples: (A) Ti-6Al-4 V (AS+*S. aureus*), (B) Ti-6Al-4 V/Nb_2_O_5_ (AS+*S. aureus*), (C) Ti-6Al-4 V (AS+*E. coli*), and (D) Ti-6Al-4 V/Nb_2_O_5_ (AS+*E. coli*) (×3000, 5 μm).

### EDS Analysis of Surface Composition

EDS measurements on the Ti-6Al-4 V/Nb_2_O_5_ sample showed signals from Nb with contributions from the substrate (Ti, Al, V)—consistent with the interaction volume sampling both the coating and underlying alloy (Table 1). Because the EDS interaction volume at the employed conditions is much larger than the ∼300 nm Nb_2_O_5_ layer (Figure 4), signals from Ti/Al/V dominate the coated sample. Consequently, the measured Nb is underquantified and does not accurately reflect the coating stoichiometry, since EDS probes in depth and captures contributions not only from the Nb_2_O_5_ film but also from the underlying Ti-6Al-4 V substrate.

**Table 1:** Atomic Concentrations (%) of the Different Predominant Chemical Elements, by EDS Analysis, of the Ti-6Al-4 V and Ti-6Al-4 V/Nb_2_O_5_ Samples

	Ti-6Al-4 V	Ti-6Al-4 V/Nb_2_O_5_
elements	atom %	atom %
Al	12.73	11.48
V	5.21	3.40
Ti	81.34	84.52
Nb		0.19

**Figure 4:**
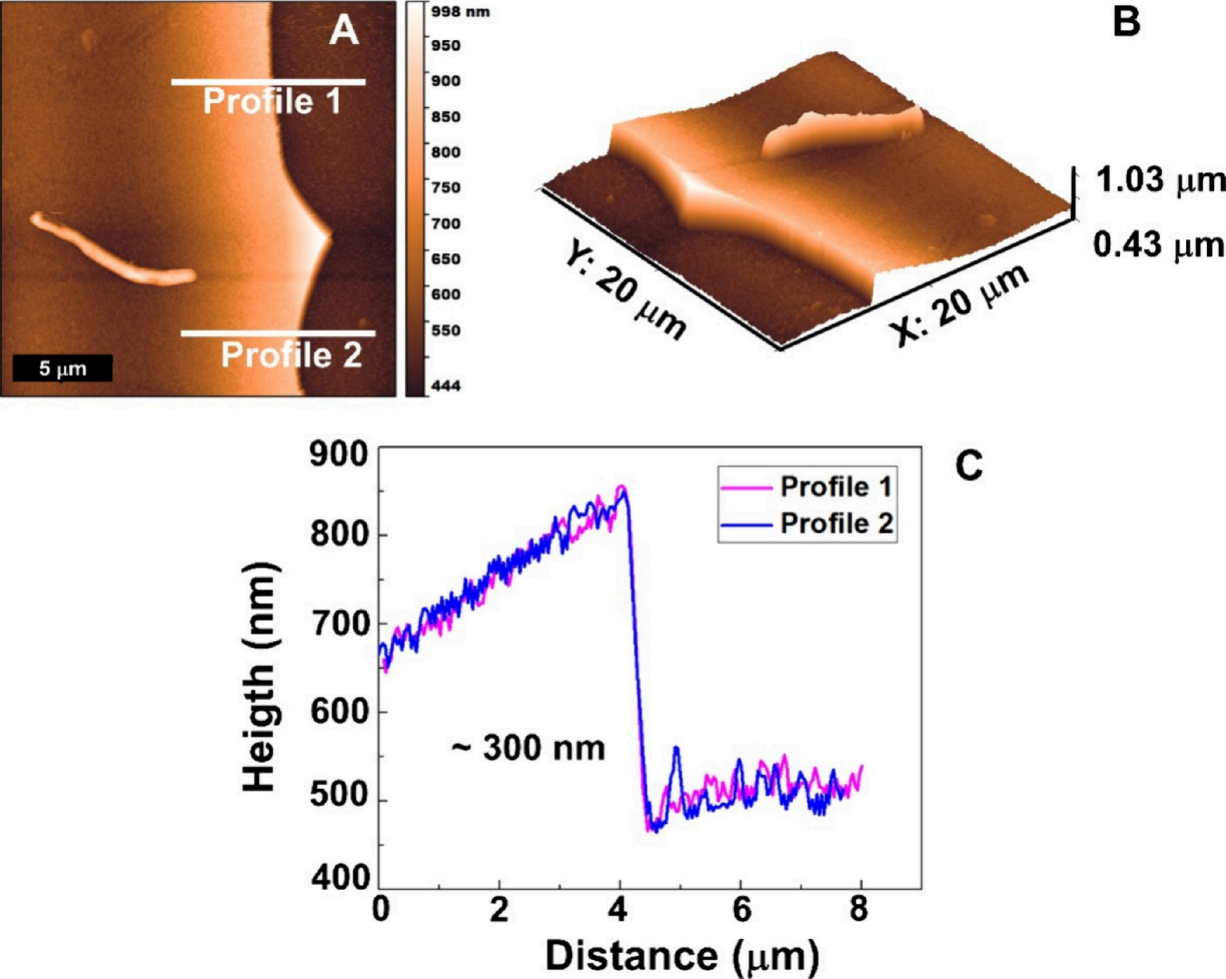
(a, b) Topographic surfaces of the Nb_2_O_5_ coating deposited on the Ti-6Al-4 V alloy using the reactive sputtering technique and (c) profilometer values in two regions indicated by traces 1 and 2 in (a).

Comparative EDS analysis of microbial interactions with Ti-6Al-4 V samples treated with AS revealed species-specific elemental profiles. Carbon was absent (0%) in *S. aureus*-contaminated samples, whereas *E. coli*-exposed samples contained 64.4% carbon (Table 4). In the *E. coli* group, atomic concentrations of Al, Ti, O, Na, Cl, K, and P were lower than those in *S. aureus*. Additionally, Mg was detected only in the Ti-6Al-4 V *S. aureus* samples, highlighting microbial-specific differences in surface interactions. Similarly, analysis of Ti-6Al-4 V/Nb_2_O_5_ samples treated with AS showed carbon absence (0%) in the *S. aureus* condition, while *E. coli* exposure resulted in 56.96% carbon (Table 2). Al, Ti, O, Na, Cl, K, and P concentrations were lower in the *E. coli* group, whereas Mg and V were undetected under either microbial condition, illustrating the combined influence of surface composition and bacterial phenotype on elemental profiles.

**Table 2:** Atomic Concentrations (%) of Predominant Chemical Elements in Ti-6Al-4 V and Ti-6Al-4 V/Nb_2_O_5_ Samples Treated with Artificial Saliva and Contaminated with *S. aureus* or *E. coli*

	Ti-6Al-4 V (AS+*S. aureus*)	Ti-6Al-4 V (AS+*E. coli*)	Ti-6Al-4 V/Nb_2_O_5_ (AS+*S. aureus*)	Ti-6Al-4 V/Nb_2_O_5_ (AS+*E. coli*)
element	atom %	atom %	atom %	atom %
Al	0.17	0.05	0.92	0.19
Ti	1.50	0.46	8.85	2.20
C		64.40		56.96
O	70.64	24.29	39.05	21.81
Na	15.18	5.57	28.82	10.62
Cl	9.59	3.86	18.35	6.36
K	0.85	0.42	1.03	0.41
P	1.71	0.86	1.74	0.99
Mg	0.18			
V	0.07			
Nb			1.24	0.46

### Surface Retention Percentage Analysis

Table 3 presents a quantitative analysis of (%) retained organic material and bacterial biofilms on Ti-6Al-4 V and Ti-6Al-4 V/Nb_2_O_5_ sample surfaces, emphasizing the effects of AS and bacterial strains (*S. aureus* and *E. coli*) under the different experimental conditions described in the Methods.

**Table 3:** Surface Coverage (%) of Retained Organic Material or Bacterial Biofilm on Ti-6Al-4 V and Ti-6Al-4 V/Nb_2_O_5_ Surfaces under Different Experimental Conditions

sample group		surface coverage area biofilm coverage (%)	observations
surface	condition	mean	surface-biofilm features
Ti-6Al-4 V	control no biofilm	91.79	homogeneous surface, low topographical retention
Ti-6Al-4 V/Nb_2_O_5_	control no biofilm	81.17	microroughness, moderate retention
Ti-6Al-4 V	*S. aureus*	40.68	lower biofilm retention, scattered adhesion sites
Ti-6Al-4 V/Nb_2_O_5_	*S. aureus*	67.42	moderate-to-high biofilm retention, more uniform coverage
Ti-6Al-4 V	*E. coli*	74.94	high biofilm coverage, patchy but extensive colonization
Ti-6Al-4 V/Nb_2_O_5_	*E. coli*	51.10	moderate adhesion, relatively more organized microcolony distribution
Ti-6Al-4 V	AS	99.83	high salivary retention, minimal surface penetration
Ti-6Al-4 V/Nb_2_O_5_	AS	85.11	lower retention, greater salivary spreading and wettability
Ti-6Al-4 V	AS and *S. aureus*	42.63	discontinuous, sparse biofilm aggregates
Ti-6Al-4 V/Nb_2_O_5_	AS and *S. aureus*	70.24	denser, more confluent biofilm with defined boundaries
Ti-6Al-4 V	AS and *E. coli*	82.0	biofilm with intermediate density and homogeneous dispersion
Ti-6Al-4 V/Nb_2_O_5_	AS and *E. coli*	85.83	uniform adhesion pattern: microcolonies with spatial distribution preserved

## Discussion

The surface morphology of biomaterials plays a crucial role in their interactions with biological environments, influencing properties such as wettability, microbial adhesion, and biofilm formation^25–29^ beyond the mechanical interaction with surfaces that are in contact with each other. In this study, SEM analysis revealed that uncoated Ti-6Al-4 V alloys exhibited a more homogeneous and smoother surface with fewer micrometric irregularities, while Ti-6Al-4 V/Nb_2_O_5_ surfaces displayed increased roughness, heterogeneity, and the presence of pronounced grooves.^1,29,30^ The incorporation of Nb_2_O_5_ coating significantly altered the microtopography, enhancing surface complexity and reactivity.^5,7^ These modifications directly affected saliva–surface interactions, as Ti-6Al-4 V samples exhibited only superficial deposition upon exposure to AS, whereas Ti-6Al-4 V/Nb_2_O_5_ surfaces demonstrated deeper salivary absorption and a more uniform appearance, likely due to increased surface energy imparted by the Nb_2_O_5_ coating.^28^ Wettability plays a key role in biofilm formation by promoting the development of a stable acquired pellicle, which modulates microbial adhesion according to species-specific affinities.^16^ This effect influences both the quantity and composition of microbial retention. The SEM findings support the hypothesis that surface energy and microtopography, particularly in Nb_2_O_5_-modified alloys, dictate early microbial interactions and biofilm formation dynamics.^31,32^ In a previously published work,^8^ we demonstrated that the reactive sputtering technique has proven advantageous for producing Nb_2_O_5_ thin films on the Ti-6Al-4 V alloy, rendering the surface more hydrophilic (Δ*G*_sws_^Total^) > 0).

AFM lift-off profiling (Figure 4A–C) shows a continuous Nb_2_O_5_ film with a step of ∼300 nm, while Ra remains essentially unchanged relative to the substrate (Ti-6Al-4 V: 84.96 ± 0.08 nm; Ti-6Al-4 V/Nb_2_O_5_: 84.34 ± 0.56 nm), indicating that the coating does not appreciably alter nanoscale roughness; thus, the biointerface differences arise primarily from surface chemistry/energy rather than topography. Consistently, contact angle data (Figure 5A) reveal lower θ on the coated surface (water ≈ 52°; formamide ≈ 48°; α-bromonaphthalene ≈ 33°), and surface free energy analysis (Figure 5B) shows a switch in (Δ*G*_sws_^Total^) from negative on Ti-6Al-4 V (−60.0 mJ m^–2^) to positive on Ti-6Al-4 V/Nb_2_O_5_ (+2.85 mJ m^–2^), evidencing increased hydrophilicity driven by a larger acid–base component. This shift mechanistically explains the deeper spreading of AS and the reduced nonspecific organic retention observed on coated samples while also being compatible with the species-dependent biofilm responses reported here (greater *S. aureus* clustering versus comparatively lower *E. coli* retention) through changes in pellicle formation and initial adhesion energetics. When exposed to *S. aureus* and *E. coli* without prior saliva treatment, Ti-6Al-4 V/Nb_2_O_5_ alloy exhibited reduced *S. aureus* colonization, while Ti-6Al-4 V/Nb_2_O_5_ surfaces displayed higher microbial retention, possibly due to enhanced mechanical anchoring from surface grooves and microcavities.^30,33^ In the presence of AS, both alloys supported denser and more structured biofilms. Saliva, rich in proteins and glycoproteins, acts as a conditioning film that facilitates *S. aureus* adhesion, consistent with its strong affinity for proteinaceous surfaces and its ability to form robust, agglomerate biofilm clusters.^15,29^

**Figure 5:**
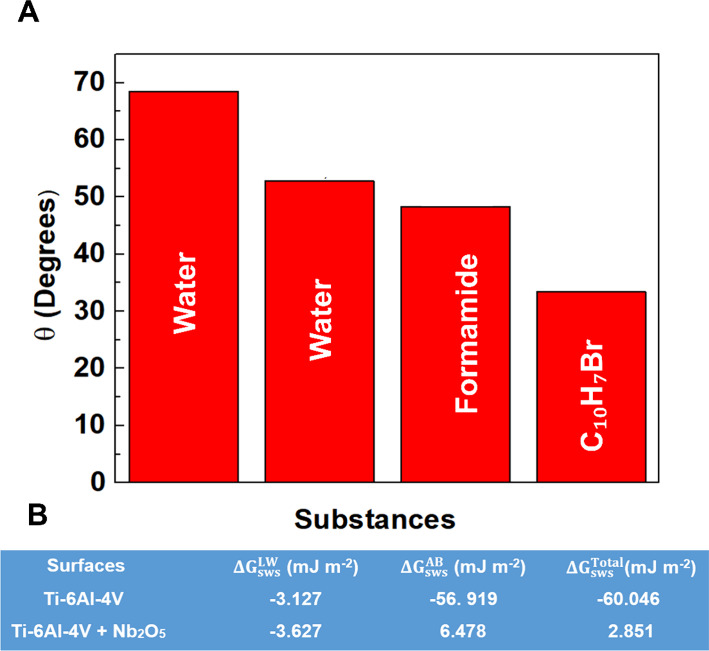
(A) Results of wettability measurements, and (B) values of polar (Δ*G*_sws_^AB^) and nonpolar compounds (Δ*G*_sws_^LW^) of the total free energy (Δ*G*_sws_^Total^) of interaction of Ti-6Al-4 V and Ti-6Al-4 V/Nb_2_O_5_.

*S. aureus* biofilms are typically dense, with an extracellular matrix rich in proteins and polysaccharides, appearing as rough dendritic structures under SEM.^18^ The complex topography of Ti-6Al-4 V/Nb_2_O_5_ further amplifies this effect, supporting organized bacterial clusters and enhancing colonization. In contrast, *E. coli* biofilms exhibited a thinner and more dispersed morphology on both surfaces.^16^ Interestingly, while Ti-6Al-4 V/Nb_2_O_5_ promoted stronger *S. aureus* adhesion, it appeared to reduce *E. coli* retention, suggesting a microorganism-dependent interaction likely governed by differences in surface charge, hydrophobicity, and the physicochemical nature of the oxide layer.^15,16,19,34^ These findings reinforce the role of surface morphology and chemistry in modulating biofilm formation, with Ti-6Al-4 V/Nb_2_O_5_ surfaces showing increased susceptibility to *S. aureus* adhesion—particularly in the presence of saliva—while *E. coli* formed thinner biofilms with reduced surface impact. This highlights a species-specific response with potential implications for selectively controlling bacterial colonization in biomedical applications.^8,28,31^

EDS analysis revealed distinct elemental signatures associated with microbial colonization, indicating species-specific interaction mechanisms with the evaluated surfaces Ti-6Al-4 V alloy samples exposed to *S. aureus* showed negligible carbon signals, implying limited organic residue accumulation.^32^ Conversely, surfaces challenged with *E. coli* presented substantial carbon content (64.4% on Ti and 56.96% on Ti-6Al-4 V/Nb_2_O_5_), suggestive of higher deposition of cellular debris or extracellular substances.^35^ These findings align with the structural differences between Gram-negative and Gram-positive bacteria; *E. coli* possesses an outer membrane enriched in lipopolysaccharides that enhances surface adhesion^36^ and carbonaceous retention, whereas *S. aureus*, with its thick peptidoglycan cell wall, demonstrated weaker adherence^37^ on unmodified Ti-6Al-4 V surfaces. This behavior corroborates previous SEM/EDS studies showing carbon-rich profiles in early *E. coli* biofilm development, often driven by fimbriae and curli-mediated attachment.^31^ Additionally, the observed elevation of sodium and chloride in *S. aureus*-exposed samples may result from interactions with salivary components or biofilm-induced ionic retention, as also noted in biofilm matrix studies where Na and P signals increased substantially with matrix development.^32^ Interestingly, trace levels of Mo and Mg were detected under the *S. aureus* condition but not in the *E. coli*-exposed samples, which may indicate microbial metabolic byproducts reacting with alloy constituents or a higher affinity for inorganic ion entrapment during *S. aureus* biofilm maturation. Taken together, these results suggest that *S. aureus* establishes more chemically interactive biofilms on both Ti-6Al-4 V and Ti-6Al-4 V/Nb_2_O_5_ surfaces, while *E. coli* tends to modulate its interaction based on surface chemistry, particularly favoring Nb_2_O_5_ coatings. This differential behavior highlights the potential of Nb_2_O_5_ coatings to modulate surface–microbe interactions in a microorganism-dependent manner, likely by altering initial adhesion dynamics and biofilm matrix composition.^21^

Threshold-based quantitative analysis further demonstrated that Ti-6Al-4 V/Nb_2_O_5_ surfaces without bacterial exposure exhibited 81.17% surface coverage within the defined gray-level interval (60–144), compared to 91.79% for uncoated Ti-6Al-4 V surfaces. This reduction in background retention suggests distinct micromorphological characteristics between the two substrates. Upon exposure to AS, uncoated Ti-6Al-4 V surfaces showed increased coverage (99.83%), reflecting enhanced adsorption of organic components, whereas Ti-6Al-4 V/Nb_2_O_5_ surfaces exhibited lower coverage (85.11%), indicating that the Nb_2_O_5_ layer reduces organic adsorption—likely due to modified surface energy and wettability.^14,18^ Following exposure to *S. aureus* and saliva, biofilm retention on Ti-6Al-4 V surfaces decreased to 42.63%, confirming the formation of well-defined, localized clusters distinct from uniform organic deposition. Under identical conditions, Ti-6Al-4 V/Nb_2_O_5_ surfaces exhibited 70.24% coverage, indicating more extensive *S. aureus* colonization. The difference in biofilm organization suggests that the Nb_2_O_5_ coating influences microbial adhesion and extracellular matrix development.^26,27,36^

Senocak et al.^27^ demonstrated that Nb_2_O_5_-rich amorphous coatings promote increased bacterial diffusion due to electrostatic interactions and surface energy profiles. Our findings indicate that surface chemistry and structure significantly modulate biofilm retention. However, while their R10 coatings (Nb_2_O_5_-dominant) facilitated *E. coli* adhesion, Ti-6Al-4 V/Nb_2_O_5_ samples in this study exhibited reduced *E. coli* coverage (51.10%) compared to uncoated Ti-6Al-4 V (74.94%) under identical conditions. This discrepancy may arise from differences in coating crystallinity, microstructure, or ion incorporation (e.g., oxynitride phases), which were not present in our sputtered oxide films.^38,39^ Notably, the Ti-6Al-4 V/Nb_2_O_5_ coatings also exhibited lower *S. aureus* biofilm retention than Ti-6Al-4 V, consistent with antibacterial effects observed in oxynitride surfaces,^27^ possibly due to surface energy modulation and reduced protein-mediated adhesion on the oxide surface. Regarding samples exposed to *E. coli*, Ti-6Al-4 V surfaces showed 82% surface coverage—nearly double that observed for *S. aureus* under equivalent conditions. This broader distribution aligns with the motility and colonization behavior of Gram-negative bacteria. The Ti-6Al-4 V/Nb_2_O_5_ surfaces exposed to *E. coli* exhibited 74.94% coverage, lower than the Ti-6Al-4 V group but with more homogeneous and spatially preserved microcolonies. These findings suggest that while the Nb_2_O_5_ coating does not inhibit *E. coli* adhesion, it may influence biofilm structure and stability.^27,28,40^

Overall, quantitative surface coverage analysis confirmed that organic and microbial retention varied according to substrate properties and experimental conditions.^41^ The uncoated and coated Ti-6Al-4 V with Nb_2_O_5_ coatings exhibited limited background retention, whereas AS significantly increased surface coverage, particularly on uncoated Ti-6Al-4 V. In microbial conditions, *S. aureus* adhesion was more pronounced on Ti-6Al-4 V/Nb_2_O_5_, whereas *E. coli* formed structured but thinner biofilms on both surfaces. These results highlight the species-specific nature of bacterial–surface interactions and demonstrate that Nb_2_O_5_ coatings modulate biofilm architecture through changes in surface chemistry and morphology.^26–28^ This reinforces the potential of Nb-based coatings to enhance biomedical materials by selectively influencing bacterial colonization.^28–33^

## Conclusions

This study demonstrates that the retention of bacterial biofilms on nanostructured Nb_2_O_5_-coated Ti-6Al-4 V surfaces is modulated by surface chemistry, microtopography, and the presence of organic substrates. The Nb_2_O_5_ coatings reduced nonspecific salivary retention and influenced bacterial adhesion in a species-dependent manner—attenuating *S. aureus* colonization while enhancing and swelling *E. coli* microcolony organization. Reactive-sputtered Nb_2_O_5_ forms a continuous ∼300 nm film on Ti-6Al-4 V; nanoscale roughness remains essentially unchanged, while wettability and surface free energy shift toward greater hydrophilicity. Under both unconditioned and saliva-conditioned assays, high-magnification SEM reveals species-dependent adhesion: *S. aureus* tends to compact clusters, whereas *E. coli* forms thinner, laterally dispersed deposits. Saliva-only controls show lower nonspecific retention on Nb_2_O_5_ than on bare Ti-6Al-4 V. Collectively, the data support a chemistry-driven—rather than topography-driven—modulation of early biointerface events. Future work will quantify viability and biomass (Live/Dead-CLSM), resolve pellicle chemistry and adsorption kinetics (XPS/ToF-SIMS), assess adhesion under flow and in mixed-species consortia, and evaluate coating integrity and durability (scratch/peel testing; SBF aging). These findings underscore the relevance of the Nb_2_O_5_ coating as a promising strategy for engineering multifunctional surfaces with tailored antimicrobial and biointeractive properties for biomedical applications.

## Experimental Section

### Materials

Ti-6Al-4 V alloy substrates were used under their as-received condition. The chemical composition (wt %) of the Ti-6Al-4 V alloy used in the present work is 0.05 N, 0.08 C, 0.015 H, 0.40 Fe, 0.20 O, 5.5–6.75 Al, 3.5–4.5 V, and Ti balance. Before the deposition process, Ti-6Al-4 V specimens were ground by using silicon carbide (SiC) abrasive papers in the sequence range 600, 800, 1200, 2400, and 4000 mesh. After the sanding process, the samples were polished with 3, 2, and 1 μm diamond paste. Following grinding, the samples were ultrasonically cleaned in distilled water and isopropyl alcohol for 10 min at ambient temperature (25 ± 1 °C). The specimens were subsequently stored in appropriate holders under clean conditions until the deposition process was initiated via a reactive sputtering technique.

### Deposition of the Nb_2_O_5_ Thin Films on the Ti-6Al-4 V Alloy Surfaces by Using the Reactive Sputtering Technique

The Nb_2_O_5_ thin films were deposited on the Ti-6Al-4 V surfaces using a reactive sputtering system, following the parameters described by Machuno et al.^42^ Key process variables included the substrate-to-target distance, deposition duration, and partial pressures of working gases. A high-purity niobium target (99.999%), supplied by Companhia Brasileira de Metallurgia e Mineração (*Brazilian Metallurgy and Mining Company*) (CBMM), was used. The sputtering atmosphere consisted of a controlled mixture of argon (99.99%) and oxygen (99.99%, White Martins), maintained at partial pressures of 5.0 and 0.5 mTorr, respectively, with an applied voltage of 440 V and a current of 140 mA. All the parameters mentioned were optimized in previous work, and further information can be obtained in the following references.^7–11,30^

### Determination of the Thickness of Nb_2_O_5_ Coating Produced via the Reactive Sputtering Technique

In previously published studies,^7–11,30^ the topography of the uncoated and coated Ti-6Al-4 V alloy was investigated using atomic force microscopy (AFM). For this purpose, a Shimadzu SPM9700 AFM, operated in phase mode, was utilized alongside cantilevers procured from NT-MDT Co. The AFM results, obtained in contact mode for the Nb_2_O_5_ thin film deposited via reactive sputtering on the Ti-6Al-4 V specimen, are illustrated in Figure 4.

The results obtained after the removal of the Kapton tape, which was used to determine the thickness of the Nb_2_O_5_ coating at various points across the surface may be seen in Figure 4a. Two profiles (represented as profiles 1 and 2) were used to delineate the interface between the Ti-6Al-4 V substrate and the Nb_2_O_5_ thin film, enabling an estimation of the film thickness, which was determined to be approximately 300 nm, as reflected in the graph presented in Figure 4c. Finally, the surface roughness of Ti-6Al-4 V and Ti-6Al-4 V/Nb_2_O_5_ was characterized, yielding comparable *R*_a_ values of 84.96 ± 0.08 and 84.34 ± 0.56 nm, respectively.^30^

### Wettability Measurements

Considering applications in the biomedical sectors, the development of comprehensive studies on material wettability becomes of paramount importance. In other words, understanding the contact behavior between liquids and biocompatible surfaces is essential for optimizing the interaction between implants, prostheses, and other biomedical devices with the biological environment. Although these details have previously been presented in an earlier publication, we believe that it is pertinent to provide additional clarifications to enhance the reader’s understanding.^8^ The wettability was assessed by measuring the contact angle (θ), defined as the angle formed between the tangent line to the liquid surface and the horizontal plane of the substrate with and without the Nb_2_O_5_ coating. According to literature,^43^ a contact angle greater than 90° indicates the absence of wetting, meaning the liquid does not spread over the solid surface, characteristic of a hydrophobic surface. Conversely, when θ is less than 90°, wetting occurs, and the liquid spontaneously spreads across the solid, indicating a hydrophilic surface. Surfaces classified as superhydrophobic typically exhibit contact angles exceeding 165° or reaching 180°, while a contact angle approaching 0° corresponds to the liquid spreading extensively and indefinitely on the solid surface. de Almeida Bino and colleagues^8^ investigated the effect of Nb_2_O_5_-based coatings on the wettability of Ti-6Al-4 V alloy in the presence of three substances: distilled water, α-bromonaphthalene (C_10_H_7_Br), and formamide (HCONH_2_), to determine the total surface free energy (Δ*G*_sws_^Total^). As reported by the authors, the application of the reactive sputtering technique resulted in a reduction of the θ values on the coated surface, measuring 33.4° for α-bromonaphthalene and 48.3° for formamide, respectively. In all cases, the contact angles remained below 90°, indicating a hydrophilic nature. When considering (Δ*G*_sws_^Total^), the authors demonstrated that the Ti-6Al-4 V alloy tends to be hydrophobic. Conversely, the Ti-6Al-4 V alloy with Nb_2_O_5_ thin films displays a more hydrophilic character, which is highly advantageous for biological applications. Figure 5a shows the results of wettability measurements, considering the Ti-6Al-4 V alloy in the presence of water, Ti-6Al-4 V/Nb_2_O_5_ in water, Ti-6Al-4 V/Nb_2_O_5_ in formamide and Ti-6Al-4 V/Nb_2_O_5_ in α-bromonapththalene determined by the Van Oss model.^8^ Figure 5b displays the values of polar (Δ*G*_sws_^AB^) and nonpolar compounds (Δ*G*_sws_^LW^) of the total free energy (Δ*G*_sws_^Total^ > 0) of interaction of Ti-6Al-4 V and Ti-6Al-4 V/Nb_2_O_5._

### Analysis of Surface Bacterial Retention Behavior

To evaluate the adhesion and microbial retention on square samples of Ti-6Al-4 V and Ti-6Al-4 V/Nb_2_O_5_, standardized of 1 cm^2^, of each material, they were previously sterilized with moist heat for 15 min at 121 °C and randomly separated into 2 groups: Ti-6Al-4 V Group (*n* = 5) and Ti-6Al-4 V/Nb_2_O_5_ Group (*n* = 5), as shown in Table 4.

**Table 4:** Analysis of the Surface Bacterial Retention Behavior in Different Groups

groups	group Ti	group Ti(Nb_2_O_5_)	description
procedures	Ti-6Al-4 V	Ti-6Al-4 V/Nb_2_O_5_	samples without artificial saliva and microorganisms
	Ti-6Al-4 V (AS)	Ti-6Al-4 V/Nb_2_O_5_ (AS)	samples with artificial saliva and without microorganisms
	Ti-6Al-4 V (*S. aureus*)	Ti-6Al-4 V/Nb_2_O_5_ (*S. aureus*)	samples without artificial saliva and with microorganisms
	Ti-6Al-4 V (AS+*S. aureus*)	Ti-6Al-4 V/Nb_2_O_5_ (AS+*S. aureus*)	samples with artificial saliva and *S. aureus* microorganisms
	Ti-6Al-4 V (AS+*E. coli*)	Ti-6Al-4 V/Nb_2_O_5_ (AS+*E. coli*)	samples with artificial saliva and *E. coli* microorganisms

### Surfaces Procedures

To evaluate the promotion of microbial retention, samples from the groups: Ti-6Al-4 V (AS); Ti-6Al-4 V/Nb_2_O_5_ (AS); Ti-6Al-4 V­(AS+*S. aureus*); Ti-6Al-4 V/Nb_2_O_5_ (AS+*S. aureus*); Ti-6Al-4 V (AS+*E. coli*); Ti-6Al-4 V/Nb_2_O_5_ (AS+*E. coli*), received a salivary film formed with AS (carboxymethyl 1%, sodium chloride 0.0084%, potassium chloride 0.12%, potassium phosphate monobasic 0.0342%, calcium chloride 0.0146%, magnesium chloride 0.0052%, Therapeutics, Manipulating Pharmacy, São Paulo, São Paulo state, Brazil). The samples were individually fully submerged in 1 mL of AS in a 24-well plate. Then, the plate was incubated for 60 min at 37 ± 1 °C, after which the samples were carefully removed, using sterile tweezers, and placed to dry for 30 min, on a sterile plate, inside the laminar flow chamber.^13^

### Microbiological Procedures

Strains of *S. aureus* (ATCC 25923) and *E. coli* (ATCC 25922) were grown separately in Brain Heart Infusion broth (Brain Heart Infusion (BHI), Kasvi, Paraná, Brazil) at 37 ± 1 °C for 24 h in a bacteriological incubator.^44^ After this period, bacterial suspensions were obtained in phosphate-buffered saline (PBS) solution (pH ∼ 7.4) at a concentration of 10^7^ CFU/mL, determined using the McFarland nephelometric scale (0.5 McFarland) for each microorganism. The samples were placed individually in a 24-well plate and were completely submerged in 1 mL of bacterial suspensions of *S. aureus* (10^7^ CFU/mL) and *E. coli* (10^7^ CFU/mL), separately, and remained in contact with the microorganisms for 24 h. After the incubation period, the samples were carefully removed and placed to dry naturally on a clean and sterile surface for 20 min, inside a laminar flow chamber. Figure 6 displays a schematic representation of the experimental protocol used to evaluate the surface bacterial activity of the Ti-6Al-4 V and Ti-6Al-4 V/Nb_2_O_5_ samples.

**Figure 6:**
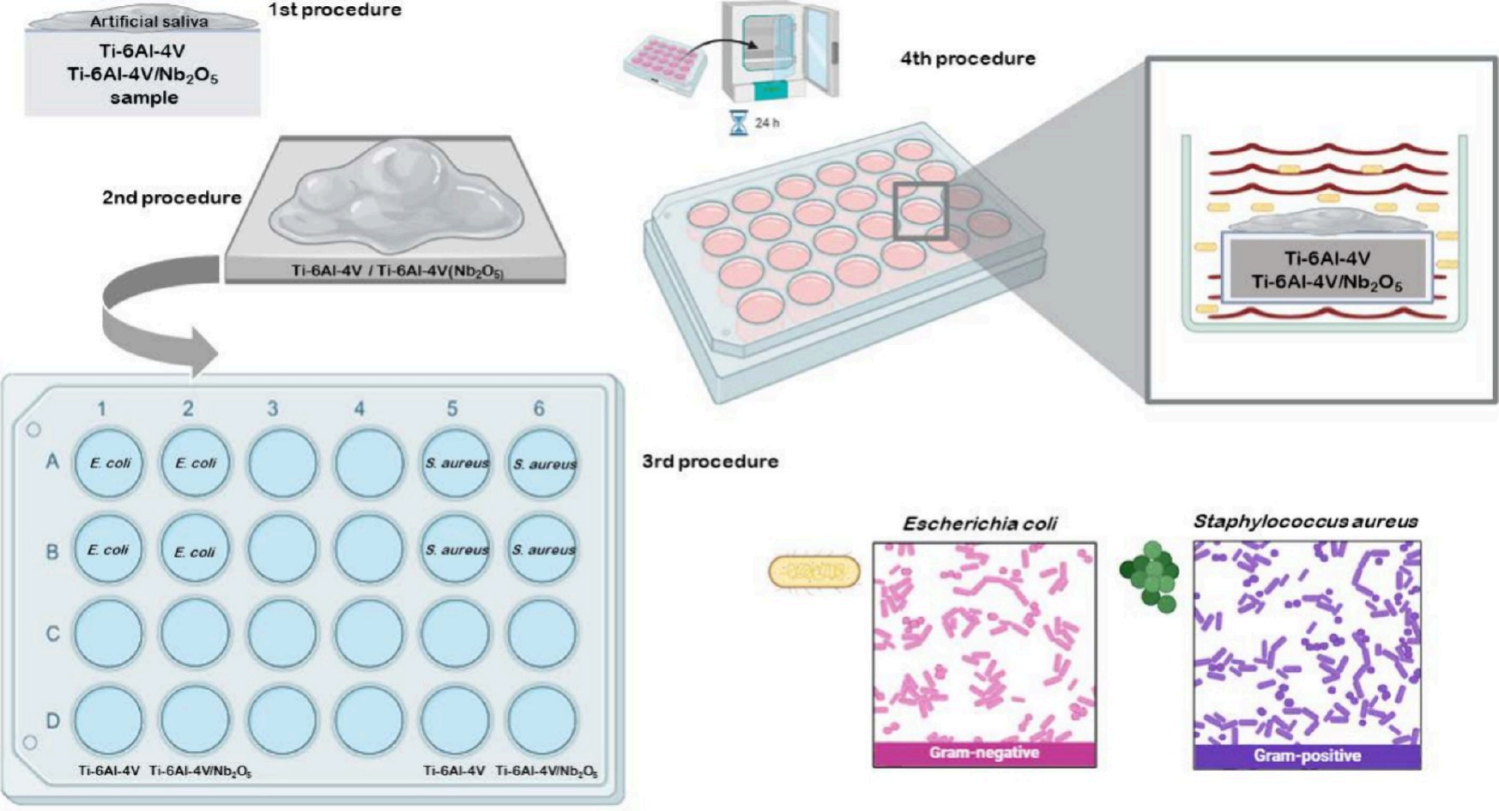
Schematic representation of the experimental protocol used to evaluate the surface bacterial activity of Ti-6Al-4 V and Ti-6Al-4 V/Nb_2_O_5_ samples. (first procedure) Surface preconditioning with deposition of thin artificial saliva film; (second procedure) sample placement into a 24-well plate; (third procedure) exposure to *E. coli* and *S. aureus* suspensions; and (fourth procedure) incubation for 24 h at 37 °C to allow bacterial adhesion and proliferation. The bacterial strains used include *S. aureus* (Gram-positive) and *E. coli* (Gram-negative). Created by the author using proprietary software.

### SEM Analysis

To characterize surface features and assess microbial retention on Ti-6Al-4 V and Ti-6Al-4 V/Nb_2_O_5_ samples subjected to distinct treatments, SEM^22–24^ imaging was performed using a Thermo Scientific UltraDry system at the SEM Laboratory of the Mackenzie Presbyterian University, São Paulo, Brazil. Samples exposed to organic substances (AS and/or bacterial suspensions) were fixed in 3% glutaraldehyde in 0.1 mol L^–1^ sodium cacodylate buffer (pH 7.4) for 12 h at 5 °C, followed by rinsing in buffer and postfixation in 2% osmium tetroxide at 50 °C for 4 h. Dehydration was carried out using a graded ethanol series (15–100%, 15 min per step). After preparation, samples were mounted on metal stubs for SEM analysis. For each sample, five photomicrographs were acquired at a standard magnification of 3000× (scale: 5 μm) from the central region to avoid edge artifacts and maximize representative surface area. Elemental mapping was performed by using energy-dispersive X-ray spectroscopy (EDS) analysis at 15.0 kV and a magnification of 100×. Spectral data were acquired in count mode, with an image resolution of 512 × 384 pixels and a pixel size of 2.36 μm. Elemental distribution maps were obtained at a resolution of 128 × 96 pixels, corresponding to a map pixel size of 9.43 μm.

### Morphological Surface Analysis

Descriptive analyses of SEM micrographs were performed by a blinded, calibrated evaluator to identify the morphological differences among experimental groups. For quantitative analysis of bacterial biofilm coverage on Ti-6Al-4 V and Ti-6Al-4 V/Nb_2_O_5_ surfaces, images were processed using Dragonfly v.2024.1 (Object Research Systems, Montreal, Canada). Pixel spacing for each image was calculated based on resolution and a known field of view (5 μm at 3000× magnification), and corresponding values were applied in Dragonfly to ensure accurate surface quantification. After image import, binary threshold segmentation was used to isolate biofilm regions based on gray-level contrast. A pixel frequency histogram was generated from each segmented image to determine the percentage of surface area covered by the biofilm. A threshold mask (Mask_Biofilm_Control) was first created from the control image using a gray-level range of 60–144. This mask was applied uniformly across all images using the Dragonfly template tool, enabling standardized segmentation of biofilm-associated features. Biofilm coverage was expressed as the ratio of pixels representing the biofilm region to the total number of pixels in the image. Control values were subtracted to account for background signals. All images were standardized for magnification and scale to ensure comparability across the conditions.

## References

[1] Yan X., Xu X., Zhou Y., Wu Z., Wei L., Zhang D. (2024). Surface electropulsing
-induced texture evolution in electron beam melted Ti-6Al-4V alloy
for biomedical application. Surf. Coat. Technol..

[2] Liu C., Yan Z., Yang J., Wei P., Zhang D., Wang Q., Zhang X., Hao Y., Yang D. (2024). Corrosion and biological
behaviors of biomedical Ti-24Nb-4Zr-8Sn alloy under an oxidative stress
microenvironment. ACS Appl. Mater. Interfaces.

[3] Ju J., Zan R., Shen Z., Wang C., Peng P., Wang J., Sun B., Xiao B., Li Q., Liu S., Yang T. (2023). Remarkable
bioactivity, bio-tribological, antibacterial, and anti-corrosion properties
in a Ti-6Al-4V-xCu alloy by laser powder bed fusion for superior biomedical
implant applications. Chem. Eng. J..

[4] Ferreira M. O. A., Mariani F. E., Leite N. B., Gelamo R. V., Aoki I. V., de Siervo A., Pinto H. C., Moreto J. A. (2024). Niobium and carbon
nanostructured coatings for corrosion protection of the 316L stainless
steel. Mater. Chem. Phys..

[5] Safavi M. S., Khalil-Allafi J., Restivo E. (2023). Enhanced in vitro immersion
behavior and antibacterial activity of NiTi orthopedic biomaterial
by HAp–Nb_2_O_5_ composite deposits. Sci. Rep..

[6] Peyghan R. A., Pouyafar V., Asghari E., Meshkabadi R. (2024). Electrophoretic
deposition of novel antibacterial and biocompatible polydopamine and
ZIF-8 hybrid composite coating on anodized Ti-6Al-4V alloy with silane
primary substrate. Surf. Interfaces.

[7] Gelamo R. V., Leite N. B., Amadeu N., Tavares M. R. P. M., Oberschmidt D., Klemm S., Fleck C., Cakir C. T., Radtke M., Moreto J. A. (2024). Exploring the Nb_2_O_5_ coating deposited on the Ti-6Al-4V alloy by
a novel GE-XANES
technique and nanoindentation load–depth. Mater. Lett..

[8] de
Almeida Bino M. C., Eurídice W. A., Gelamo R. V., Leite N. B., da Silva M. V., de Siervo A., Pinto M. R., de Almeida
Buranello P. A., Moreto J. A. (2021). Structural and morphological characterization
of Ti-6Al-4V alloy surface functionalization based on Nb_2_O_5_ thin film for biomedical applications. Appl. Surf. Sci..

[9] Moreto J. A., Gelamo R. V., da Silva M. V. (2021). New insights of Nb2O5-based
coatings on the 316L SS surfaces: enhanced biological responses. J. Mater. Sci: Mater. Med..

[10] Nascimento J. P. L., Teixeira G. T. L., Obata M. M. S., da
Silva M. V., de Oliveira C. J. F., Silva L. E. A., Gelamo R. V., Slade N. B. L., Moreto J. A. (2023). Influence of Reactive Sputtering-Deposited
Nb2O5 Coating On the Ti-6Al-4V Alloy Surfaces: Biomineralization,
Antibacterial Activity, and Cell Viability Tests. Mater. Res..

[11] Silva T., Ferreira M., Nascimento J., Pietro L., Neto L. C., Moreira H., Pereira L., Leite N., Gelamo R., Moreto J. A. (2022). Development of a
low-cost ball-on-flat linear reciprocating
apparatus: Test validation using Ti-6Al-4V and Ti-6Al-4V/Nb_2_O_5_ coatings. J. Mater. Sci. Technol.
Res..

[12] Instituto Brasileiro de Mineração (IBRAM). Plano Estadual da Mineração—Diagnóstico do Setor Mineral de Minas Gerais, 2021. Available at: https://ibram.org.br/wp-content/uploads/2021/02/PEM-MG.pdf.

[13] Brito A. C. M., Bezerra I. M., Borges M. H. S., Silva R. O., Gomes-Filho F. N., Almeida L. F. D. (2020). Adhesion of monospecies biofilms
from *Streptococcus
mutans* and *Candida albicans* to different
surfaces of conventional composite resins and bulk fill. Rev. Odontol. UNESP.

[14] Macartney R. A., Das A., Imaniyyah A. G., Fricker A. T., Smith A. M., Fedele S., Roy I., Kim H. W., Lee D., Knowles J. C. (2025). In vitro and ex
vivo models of the oral mucosa as platforms for the validation of
novel drug delivery systems. J. Tissue Eng..

[15] Lu Y., Cai W.-J., Ren Z., Han P. (2022). The role of staphylococcal
biofilm on the surface of implants in orthopedic infection. Microorganisms.

[16] Nikam M., Mane S., Jadhav S. (2025). Influence of micro-textures
on wettability and antibacterial behavior of titanium surfaces against
S. aureus and E. coli: In vitro studies. Int.
J. Interact. Des. Manuf..

[17] Civantos A., Martínez-Campos E., Ramos V., Elvira C., Gallardo A., Abarrategi A. (2017). Titanium coatings and surface modifications:
Toward clinically useful bioactive implants. ACS Biomater. Sci. Eng..

[18] Jahanmard F., Dijkmans F. M., Majed A., Vogely H. C., van der
Wal B. C. H., Stapels D. A. C., Ahmadi S. M., Vermonden T., Yavari S. A. (2020). Toward antibacterial coatings for personalized implants. ACS Biomater. Sci. Eng..

[19] Ramachandran, B. ; Muthuvijayan, V. Surface engineering approaches for controlling biofilms and wound infections. In Introduction to Biofilm Engineering; American Chemical Society: Washington, DC, 2019; 1323, 101–123.

[20] Safari-Gezaz M., Parhizkar M. (2024). Effect of ionic liquid as a surfactant in hydroxyapatite
coatings for improvement corrosion resistance of Ti-6Al-4V substrates
for implant applications. Heliyon.

[21] Qu X., Yang H., Jia B., Wang M., Yue B., Zheng Y., Dai K. (2021). Zinc alloy-based
bone internal fixation
screw with antibacterial and anti-osteolytic properties. Bioact. Mater..

[22] Fischer E. R., Hansen B. T., Nair V., Hoyt F. H., Schwartz C. L., Dorward D. W. (2024). Scanning electron
microscopy. Curr. Protoc..

[23] Porto R., Mengarda A. C., Cajas R. A., Salvadori M. C., Teixeira F. S., Arcanjo D. D. R., Siyadatpanah A., Pereira M. L., Wilairatana P., Moraes J. (2021). Antiparasitic properties
of cardiovascular agents against human intravascular parasite *Schistosoma mansoni*. Pharmaceuticals.

[24] Xavier R. P., Mengarda A. C., Silva M. P., Roquini D. B., Salvadori M. C., Teixeira F. S., Pinto P. L., Morais T. R., Ferreira L. L. G., Andricopulo A. D., de Moraes J. (2020). H_1_-antihistamines as antischistosomal drugs: *In vitro* and *in vivo* studies. Parasites
Vectors.

[25] Eduok U. (2020). Niobia nanofiber-reinforced
protective niobium oxide/acrylate nanocomposite coatings. ACS Omega.

[26] Safavi M. S., Walsh F. C., Visai L., Khalil-Allafi J. (2022). Progress in
niobium oxide-containing coatings for biomedical applications: A critical
review. ACS Omega.

[27] Senocak T. C., Ezirmik K. V., Aysin F., Simsek Ozek N., Cengiz S. (2021). Niobium-oxynitride coatings for biomedical
applications:
Its antibacterial effects and *in-vitro* cytotoxicity. Mater. Sci. Eng., C.

[28] Corado H. P. R., de Souza Soares F. M., Barbosa D. M., Lima A. M., Elias C. N. (2022). Titanium coated with graphene and niobium pentoxide
for biomaterial applications. Int. J. Biomater..

[29] Pradhan D., Wren A. W., Misture S. T., Mellott N. P. (2016). Investigating
the
structure and biocompatibility of niobium and titanium oxides as coatings
for orthopedic metallic implants. Mater. Sci.
Eng., C.

[30] Ferreira M. O. A., Wolf W., Gelamo R. V., Slade N. B. L., Galo R., Jasinevicius R. G., Moreto J. A. (2025). Advances in enhancing the wear performance
of Ti-6Al-4V biomedical alloy through Nb_2_O_5_ coating. Materials.

[31] Ballén V., Cepas V., Ratia C., Gabasa Y., Soto S. M. (2022). Clinical *Escherichia coli*: From biofilm formation to new antibiofilm
strategies. Microorganisms.

[32] Rimal B., Chang J. D., Liu C., Kim H., Aderotoye O., Zechmann B., Kim S. J. (2024). Scanning electron
microscopy and
energy-dispersive X-ray spectroscopy of *Staphylococcus aureus* biofilms. ACS Omega.

[33] Rădulescu R., Meleşcanu Imre M., Ripszky A., Rus F., Popa A., Moisa M., Funieru C., Ene R., Pituru S. (2024). Exploring the broad
spectrum of titanium–niobium
implants and hydroxyapatite coatings—A review. Materials.

[34] Quelemes P. V., Perfeito M. L., Guimarães M. A., dos Santos R. C., Lima D. F., Nascimento C., Silva M. P., Soares M. J., Ropke C. D., Eaton P., de Moraes J., Leite J. R. (2015). Effect of neem (*Azadirachta indica* A. Juss) leaf extract on resistant *Staphylococcus aureus* biofilm formation and *Schistosoma mansoni* worms. J. Ethnopharmacol.

[35] Bao S., Sun S., Li L., Xu L. (2023). Synthesis and antibacterial
activities
of Ag-TiO_2_/ZIF-8. Front. Bioeng.
Biotechnol..

[36] Pleskova S. N., Bezrukov N. A., Bobyk S. Z., Gorshkova E. N., Novikov D. V. (2024). Pathogenic *Escherichia coli* change
the adhesion between neutrophils and endotheliocytes in the experimental
bacteremia model. Microb. Cell.

[37] Dramsi, S. ; Bierne, H. Spatial organization of cell wall-anchored proteins at the surface of Gram-positive bacteria. In Current Topics in Microbiology and Immunology; Springer: Berlin, Heidelberg, 2017; 404, 177–201. 27025379 10.1007/82_2016_4

[38] Tardelli J. D. C., Bagnato V. S., Reis A. C. D. (2023). Bacterial adhesion strength on titanium
surfaces quantified by atomic force microscopy: A systematic review. Antibiotics.

[39] Puzas V. M. V., Carter L. N., Schröder C., Colavita P. E., Hoey D. A., Webber M. A., Addison O., Shepherd D. E. T., Attallah M. M., Grover L. M., Cox S. C. (2022). Surface
free energy dominates the
biological interactions of postprocessed additively manufactured Ti-6Al-4V. ACS Biomater. Sci. Eng..

[40] Fais L. M. G., de Sales Leite L., Reis B. A. D., Ribeiro A. L. R., Vaz L. G., Klein M. I. (2021). Microbial
adhesion and biofilm formation
on bioactive surfaces of Ti-35Nb-7Zr-5Ta alloy created by anodization. Microorganisms.

[41] Li H., Ding Y., Hu X., Li W., Ding Z. (2024). A comparative
study of TiO_2_, Ta_2_O_5_ and Nb_2_O_5_ coated Ti-6Al-4V titanium alloy for biomedical applications. Ceram. Int..

[42] Machuno L. G. B., Lima A. B., Buso R. R., Abdanur R. M. F., Rangel E. C., Gelamo R. V. (2016). Desenvolvimento
e avaliação de uma fonte
DC de alta tensão para utilização em sistema
de deposição de filmes finos por pulverização
catódica. Matéria (Rio J.).

[43] Luz A. P., Ribeiro S., Pandolfelli V. C. (2008). Uso da molhabilidade na investigação
do comportamento de corrosão de materiais refratários. Cerâmica.

[44] Silva, P. I. B. P. ; Saleh, M. A. K. ; Baptista, A. ; Honorato, D. ; Campos, S. ; Nunez, S. C. ; Navarro, R. S. Antimicrobial Photodynamic Therapy with Methylene Blue and Urea in Escherichia coli and Staphylococcus aureus. In IX Latin American Congress on Biomedical Engineering and XXVIII Brazilian Congress on Biomedical Engineering. CLAIB/CBEB 2022: IFMBE Proceedings, Vol. 4: Clinical Engineering and Health Technologies; Marques, J. L. B. ; Rodrigues, C. R. ; Suzuki, D. O. H. ; Marino Neto, J. ; García Ojeda, R. , Eds.; Springer: Cham, 2024; 101; 349–356.

